# A Rare Case of Gould Syndrome Presenting With Gastrointestinal Bleeding in a Pediatric Patient

**DOI:** 10.7759/cureus.114614

**Published:** 2026-08-16

**Authors:** Nelson Gonzalez, Peyton Pearrow, Sandeep K Puri

**Affiliations:** 1 Internal Medicine, University of Florida, Gainesville, USA; 2 Pediatrics, University of South Carolina, Columbia, USA; 3 Pediatric Gastroenterology, Prisma Health, Columbia, USA

**Keywords:** col4a1 mutation, gastrointestinal bleeding, gould syndrome, pediatric gastrointestinal bleeding, symptomatic anemia

## Abstract

Gould syndrome, caused by COL4A1/COL4A2 mutations, is a rare multisystem disorder characterized by basement membrane defects leading to vascular fragility, cerebral small vessel disease, seizures, and renal involvement. While vascular complications are well-described, gastrointestinal bleeding remains exceedingly rare. We describe a four-year-old female with a known diagnosis of Gould syndrome who presented with hemodynamically significant hematochezia. Colonoscopy demonstrated tortuous submucosal vascular lesions consistent with colonic varices in the sigmoid colon, which were treated with bipolar probe electrocautery; an additional non-bleeding varix was noted at the hepatic flexure. Computed tomography (CT) angiography and time-resolved imaging of contrast kinetics magnetic resonance imaging (TRICKS MRI) revealed abnormal venous structures in the mesentery and bowel without evidence of arteriovenous malformation or portal-systemic shunting, congruent with endoscopic findings. There was no clinical or radiographic evidence of portal hypertension. This case expands the phenotypic spectrum of Gould syndrome by providing detailed evidence of gastrointestinal venous abnormalities and highlights gastrointestinal bleeding as a potential underrecognized manifestation of COL4A1/2-related disease.

## Introduction

Gould syndrome is a rare autosomal dominant disorder caused by mutations in the COL4A1 or COL4A2 genes, which are two of six genes encoding the alpha chains of type IV collagen [1]. Type IV collagen is a nonfibrillar collagen with a triple helix structure that forms pliable sheets and is a key structural component of basement membranes, including vascular endothelium [2,3]. The basement membrane zone provides structural support and serves as an important site of intermolecular cross-linking and cellular networking [2]. Mutations in the COL4A1/2 genes impact basement membrane integrity and organization as well as intercellular signaling, and cytotoxic gene products are thought to contribute to tissue damage [3,4].

Given the ubiquitous distribution of type IV collagen, affected individuals develop multisystem involvement, most commonly involving the cerebral vasculature, eyes, kidneys, and muscles. Clinical manifestations include small vessel disease, pre- and postnatal intracerebral hemorrhage, seizures, cataracts, and nephropathy [1,4]. Clinical phenotypes of patients with COL4A1/2 mutations vary widely; therefore, it is reasonable to conclude that this condition may be underdiagnosed and that unrecognized phenotypes may exist [3,4]. About 137 cases globally are represented in the literature [4]. Although vascular fragility is a hallmark of COL4A1/2-related disorders, extracerebral hemorrhagic manifestations are less frequently described [5]. Gastrointestinal involvement, in particular, is not currently recognized as a core feature of Gould syndrome [1,6]. A recent review identified only one reported case of gastrointestinal bleeding with limited clinical detail, suggesting that the full clinical spectrum of this disorder remains incompletely defined [5].

We present a pediatric case of Gould syndrome complicated by significant gastrointestinal bleeding. The patient was diagnosed with a de novo COL4A1 mutation at nine months of age after an incidental finding of periventricular white matter calcifications on head CT scan. Although she received appropriate neurological, ophthalmologic, and renal screenings, there was no initial concern for gastrointestinal pathology. At the age of four years, she presented for hematochezia and symptomatic anemia. Comprehensive endoscopy and multimodal cross-sectional imaging revealed colonic varices in the absence of portal hypertension, a finding that, to our knowledge, has not been previously reported in Gould syndrome. This case highlights a potential underrecognized manifestation of COL4A1/2-related disease and raises important considerations for clinical evaluation and surveillance.

## Case presentation

A four-year-old female with known Gould syndrome and oculocutaneous albinism presented to our institution with frank rectal bleeding on a background of hematochezia several months prior. The patient had a history of periventricular white matter calcifications incidentally discovered on a head CT scan obtained at the age of nine months following an accidental fall. Workup for causative prenatal infection was negative. Subsequent genetic testing revealed a de novo pathogenic change in the COL4A1 gene, leading to the diagnosis of Gould syndrome. A separate gene mutation responsible for her albinism was also identified. Pediatric neurosurgery determined there was no concern for cerebral hemorrhage; however, given that Gould syndrome patients are at increased risk for intracranial bleeding, the patient’s parents were counseled on monitoring for signs of seizure or stroke. The patient was followed by ophthalmology and received periodic renal function testing and a renal ultrasound, which were unremarkable. She also received physical and speech therapy for mild developmental delays. At the age of two years, she had microcytic anemia attributed to dietary iron deficiency and received iron supplementation.

The patient was brought to the emergency department after her mother noticed bright red blood in her underwear. She previously had blood-streaked stools, which were attributed to constipation for which she received a stool softener. On arrival, the patient’s vital signs were notable for tachycardia (heart rate = 104), tachypnea (respiratory rate = 30), hypotension (blood pressure = 91/38), and oxygen saturation of 96% on room air. In the emergency department, she experienced a syncopal episode. Laboratory evaluation revealed a hemoglobin level of 8.2 g/dL (11.5-14.5 g/dL) [7]. Prothrombin time (PT) was normal, and activated partial thromboplastin time (aPTT) was slightly elevated. She subsequently received fluid resuscitation. Her hemoglobin decreased to 7.3 g/dL three hours after presentation and to 6.5 g/dL after eight hours, prompting a blood transfusion at 10 cc/kg, after which hemoglobin improved to 8.1 g/dL.

**Figure 1:**
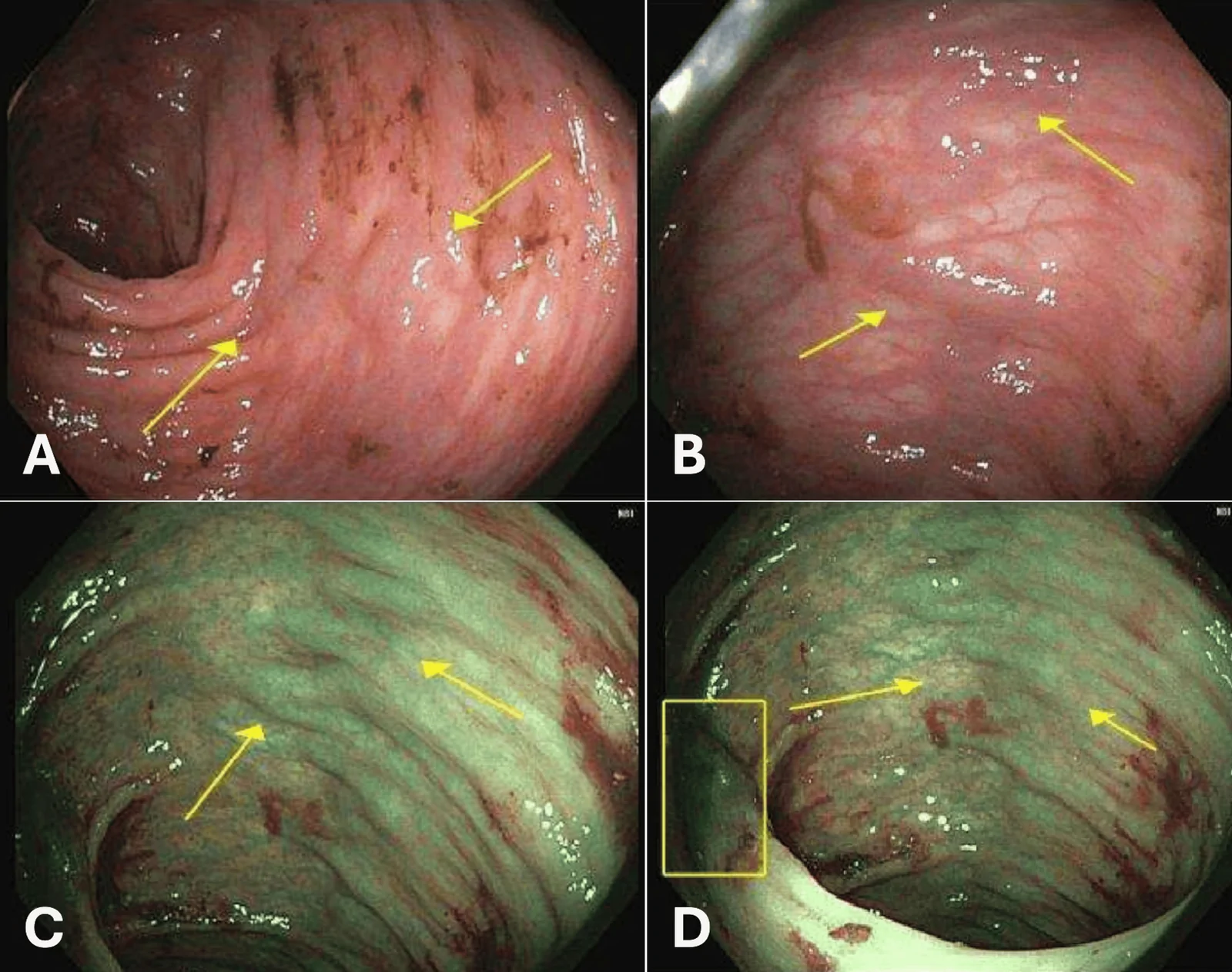
A 3-mm nonbleeding colonic varix was found at the hepatic flexure. (A) White-light endoscopic view of the varix (yellow arrows). (B) White-light view from an alternate angle demonstrating the varix within the colonic lumen (yellow arrows). (C) Narrow-band imaging* view highlighting the varix (yellow arrows). (D) Narrow-band imaging view from a different perspective demonstrating the extent of the varix (yellow arrows). * Narrow-band imaging uses specific wavelengths to visualize blood vessels and surface features on mucosa.

**Figure 2:**
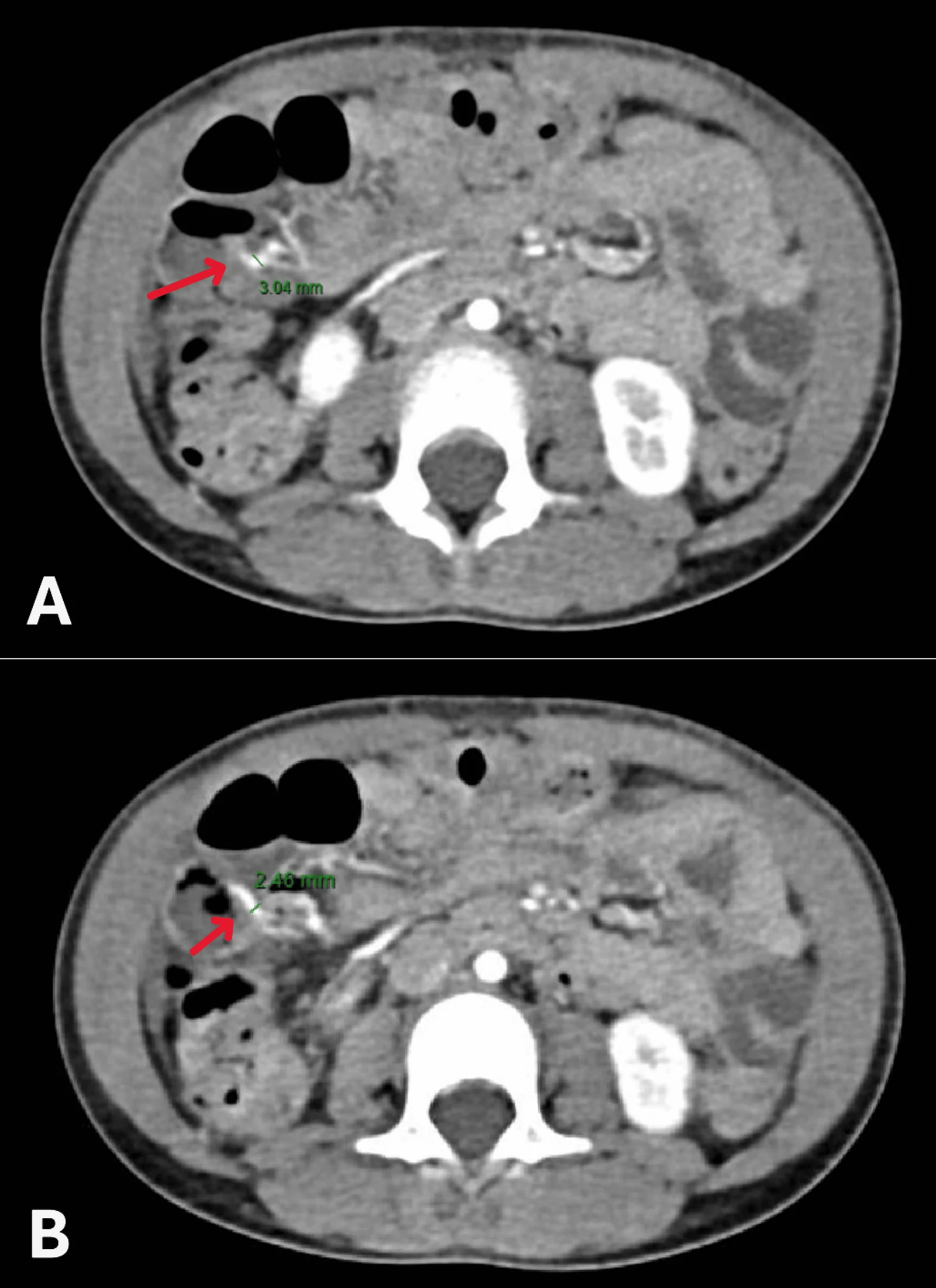
Contrast-enhanced CT angiogram of the abdomen and pelvis demonstrating prominent mesenteric vessels in the right abdomen. (A) Axial CT angiogram demonstrating prominent enhancing vessels in the right abdomen, consistent with the hepatic flexure varix identified on colonoscopy (red arrow). (B) Adjacent axial CT angiogram demonstrating continuity of the varix (red arrow).

An esophagogastroduodenoscopy (EGD) was performed but revealed no active sources of bleeding. A colonoscopy showed vascular lesions consistent with varices in the sigmoid colon, treated with bipolar probe electrocautery. Additionally, a hepatic flexure varix was noted in the right colon (Figures 1A, 1B). To further evaluate for a possible small bowel source, a capsule endoscopy was performed, but no abnormalities were detected. CT angiography revealed abnormal venous branches in the bowel and mesentery, particularly in the right abdomen, corresponding to the location of the hepatic flexure varix seen on colonoscopy (Figure 2). Time-resolved imaging of contrast kinetics (TRICKS) MRI angiography further characterized these findings, showing prominent vascular structures in the right mesentery and bowel, but without evidence of portal-to-systemic venous shunting. Vascular surgery was consulted, but no immediate action was recommended given that her anemia was stable at 8.7 g/dL (11.5-14.5 g/dL) [7].

After colonoscopy and electrocautery, the patient had no further bleeding events and tolerated oral intake. Stool softeners were restarted to minimize risk of trauma to the colonic mucosa. Given the complexity of her vascular findings and the potential association with her underlying connective tissue disorder, the patient was transferred to a tertiary care hospital for specialized management by a multidisciplinary team. Further evaluation there included a second colonoscopy, which visualized nonbleeding engorged blood vessels, but no intervention was performed. Tranexamic acid 10 mg was administered preoperatively and then postoperatively every eight hours. The patient’s bowel regimen was continued. Subsequent hemoglobin measurements were normal, and no other bleeding episodes were reported.

## Discussion

This case provides evidence supporting gastrointestinal vascular involvement as a manifestation of Gould syndrome. Our patient developed significant hematochezia resulting in symptomatic anemia, with endoscopic and radiographic findings demonstrating venous vascular abnormalities within the colon and mesentery.

The vascular lesions identified on colonoscopy were consistent with varices, characterized by dilated, tortuous submucosal veins. These findings were supported by imaging demonstrating abnormal venous structures without early arterial filling or a defined nidus, arguing against arteriovenous malformations. It is important to note that while rectal varices are common in children, true colonic varices in children are extremely rare [8]. When present, they are almost always due to portal hypertension [9]. In a prospective analysis of children with extrahepatic portal venous obstruction, 75% had rectal varices; only 3.7% had colonic varices [9]. Only a few cases of varices in the absence of portal hypertension are available in the literature [10]. This patient had no clinical, laboratory, or imaging evidence of portal hypertension, including the absence of portal-systemic shunting on MRI, suggesting that these lesions are unlikely to be secondary to elevated portal pressures and are more likely attributable to an intrinsic vascular defect related to the underlying collagen disorder. The patient’s differential diagnoses and findings are summarized in Table 1.

**Table 1:** Expected imaging and endoscopic findings for the differential diagnosis of colonic vascular lesions. In our patient with colonic varices without portal hypertension, we found focal dilated/tortuous mesenteric vessels corresponding to her endoscopic lesion; absence of portal hypertension, portal venous obstruction, generalized portosystemic collateralization, and arteriovenous malformation nidus/arteriovenous shunting. TRICKS: time-resolved imaging of contrast kinetics.

Differential diagnosis	CT angiogram	TRICKS MRI	Endoscopy
Arteriovenous malformation (AVM)	Serpiginous vessels with an arterial supply, vascular nidus, and/or early venous drainage	Arterialized vascular nidus with early venous drainage	Localized vascular-appearing lesion
Colonic varices with portal hypertension	Dilated/tortuous colonic or mesenteric veins with evidence of portal hypertension and/or portosystemic collaterals; no AVM nidus	Dilated venous structures with portosystemic collateralization; no arterialized nidus	Bluish, serpiginous submucosal veins
Colonic varices without portal hypertension	Focal dilated/tortuous colonic or mesenteric veins without evidence of portal hypertension or AVM	Focal venous dilation without generalized portosystemic collateralization or arterialized nidus	Bluish, serpiginous submucosal veins

Type IV collagen is a critical structural component of basement membranes throughout the vasculature, including the gastrointestinal tract [11]. Mutations in COL4A1/2 are known to result in vessel wall fragility and abnormal vascular architecture, most prominently in the cerebral circulation [6]. The presence of similar abnormalities in the gastrointestinal vasculature is biologically plausible but likely unrecognized. This case supports the concept that venous dilation and fragility within the gastrointestinal tract may represent an extension of the systemic vasculopathy seen in Gould syndrome.

Alternatively, liver cysts can present with symptoms of portal hypertension, although this requires significant cyst burden [12]. Liver cysts have been a rare but consistently reported manifestation of Gould syndrome [13]. Although our patient did not present with liver lesions or any radiographic evidence of portal hypertension, future cases could present this as a cause of gastrointestinal bleeding. While our case points to the vascular abnormalities present in Gould syndrome as the culprit for the bleeding, alternative mechanisms could still be possible. In fact, only one prior case of gastrointestinal bleeding in a patient with Gould syndrome has been reported, and it lacked detailed endoscopic and imaging characterization [5]. This report adds to the literature by providing a comprehensive description of both luminal and cross-sectional findings, strengthening the association between COL4A1/2 mutations and gastrointestinal vascular abnormalities.

This case has several clinical implications. Clinicians should consider gastrointestinal vascular lesions in patients with Gould syndrome who present with anemia or hematochezia, even in the absence of portal hypertension. Additionally, unexplained gastrointestinal bleeding with atypical vascular findings may warrant consideration of an underlying connective tissue or genetic vascular disorder such as COL4A1/2-related disease. Awareness of potential vascular fragility may also influence endoscopic management strategies and procedural risk assessment in these patients.

Currently, there are no formal guidelines for gastrointestinal screening in patients with Gould syndrome, and existing recommendations focus on neurologic, ophthalmologic, renal, and cardiovascular monitoring [14]. If further cases confirm GI involvement, incorporation of gastrointestinal evaluation into multidisciplinary surveillance strategies may be warranted.

As a single case report, this study cannot establish causation or define prevalence. However, it contributes to the expanding phenotypic spectrum of Gould syndrome and underscores the need for further investigation into gastrointestinal manifestations of COL4A1/2-related disorders.

## Conclusions

From a clinical perspective, this case highlights the importance of considering gastrointestinal vascular lesions in patients with Gould syndrome who present with unexplained anemia, hematochezia, or other forms of gastrointestinal bleeding. Awareness of these manifestations may prompt earlier endoscopic and radiologic evaluation, allowing for appropriate management, closer surveillance, and prevention of recurrent bleeding and related complications. In our patient, bleeding colonic varices were diagnosed via a combination of endoscopy and multimodal imaging. Hemostasis was achieved with electrocautery and tranexamic acid. The patient is now maintained on a bowel regimen to prevent mucosal trauma and monitored with periodic laboratory studies; however, patients such as ours could benefit from screening and management guidelines.

Additionally, this case adds to the limited literature on gastrointestinal involvement in Gould syndrome by providing detailed clinical, endoscopic, and imaging evidence of colonic varices in the absence of portal hypertension. These findings suggest that gastrointestinal vascular abnormalities may represent an unrecognized manifestation of COL4A1/2-related disease. Increased awareness and additional case reports are needed to better define the prevalence, underlying mechanisms, and clinical significance of gastrointestinal involvement, which may ultimately inform future screening strategies and management recommendations.
